# The complete chloroplast genome of pioneering plant *Cyperus iria* L. (Cyperaceae) in ecological restoration

**DOI:** 10.1080/23802359.2021.1908865

**Published:** 2021-04-08

**Authors:** Ling-ling Yang, Jun-qi Niu, Wen-wei Tang

**Affiliations:** a Guangxi Key Laboratory of Agric-Environment and Agric-Products Safety, Agricultural College, Guangxi University, Nanning, PR China; b Guangxi Key Laboratory of Agricultural Resources Chemistry and Biotechnology, Yulin Normal University, Yulin, PR China

**Keywords:** *Cyperus iria*, complete chloroplast genome, phylogenetic analysis

## Abstract

*Cyperus iria* L. is an annual weed of the family Cyperaceae, which plays an important role in the environmental remediation of uranium contaminate. Here, the complete chloroplast (cp) genome of *Cyperus iria* has been reconstructed from the complete genome Illumina sequencing data. The complete cp genome was 185,697 bp in length, containing a large single copy region (LSC) of 99,360 bp and a small single copy region (SSC) of 10,267 bp, which were separated by a pair of 38,035 bp inverted repeat regions (IRs). The cp genome contained 135 genes, including 89 protein-coding genes (PCGs), eight *rRNA* genes, and 38 *tRNA* genes. The cp genome has a GC content of 33.16%. Further, the phylogenetic analysis showed a strong sister relationship with *Cyperus rotundus.*

*Cyperus iria* is a troublesome weed in rice production and actively adapts to ecological niches (Jiang et al. 2018). As a uranium tolerant plant, its discovery provides a new resource for exploring the remediation of uranium-contaminated environment and the mechanism of uranium absorption and tolerance in plants (Nie et al. 2015). Here, we assembled and annotated the complete cp genome of *C. iria* using high-throughput sequencing technology, which will be helpful for further studies on the function of cp genes and its phylogenetic evolution.

The fresh leaves of *C. iria* were collected from a single individual in Guigang city (Located at 109°11′ to 110°39′ east longitude, 22°39′ to 24°2′ north latitude), Guangxi Province, China. The voucher specimens of *C. iria* were deposited at the herbarium of Guangxi University (https://www.gxu.edu.cn/, Wen-wei Tang, wenweitg@163.com) under the voucher number: 20200701Y01, and DNA samples were properly stored at laboratory of Agric-Environment and Agric-Products Safety, Guangxi University. Genomic DNA was extracted from the fresh leaves according to a modified CTAB method (Doyle JJ and Doyle JL 1987), and constructed the libraries with an average length of 350 bp using the NexteraXT DNA Library Preparation Kit (Illumina, San Diego, CA). Then the libraries were sequenced on Illumina Novaseq 6000 platform, 4.35 Gb clean data were de novo assembled using SPAdes version 3.11.0 software (St Petersburg, Russia) (Bankevich et al. 2012). Finally, the complete cp genome of *C. iria* was annotated using the PGA software (KunMing, China) (Qu et al. 2019). The physical map of *C. iria* was visualized using OGDRAW online tool (Lohse et al. 2013). The cp genome sequence of *C. iria* has been deposited into the GenBank (MW123056).

The cp genome of *C. iria* is a circular DNA molecule with 185,697 bp in length, containing a large single copy region (LSC) of 99,360 bp and a small single copy region (SSC) of 10,267 bp, which were separated by a pair of 38,035 bp inverted repeat regions (IRs). The total GC content of the cp genome is 33.16%. The cp genome harbors 135 functional genes, including 89 protein-coding genes (PCGs), 38 *tRNA* genes, and eight *rRNA* genes. Among them, 49 are involved in photosynthesis, and 76 genes are involved in selfreplication. Moreover, among all the PCGs, 15 genes contain one intron, whereas *ycf3* gene has two introns. The *rps12* gene which encodes the 40S ribosomal protein S12, was trans-spliced.

To determine the phylogenetic position of *C. iria*, 12 complete cp genome sequences were aligned by MAFFT version 7.471 (Yamadaoka, Suita, Osaka, Japan) (Kazutaka et al. 2002), and we used the MEGA version 7.0 (Auckland, New Zealand) (Kumar et al. 2016) to construct a phylogenetic tree with a bootstrap value of 1000. The maximum-likelihood (ML) phylogenetic analysis (Figure 1) showed the position of *C. iria* was situated as the sister of *Cyperus rotundus* in Cyperaceae. Our findings provide valuable information on the genetic diversity research of *C. iria* and enrich the resources of cp genomes in pioneering plants.

**Figure 1.**
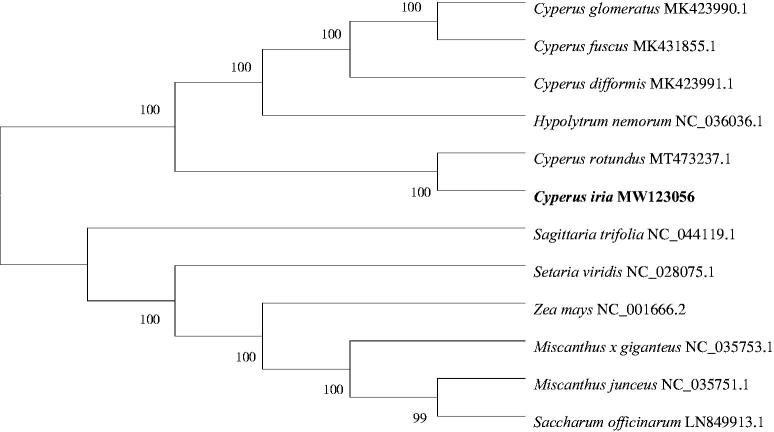
Phylogenetic tree of 12 species based on the maximum-likelihood analysis of the complete chloroplast genome sequences using 1000 bootstrap replicates.

## Data Availability

The genome sequence data that support the findings of this study are openly available in GenBank of NCBI at (https://www.ncbi.nlm.nih.gov/) under the accession no.MW123056. The associated BioProject, SRA, and Bio-Sample numbers are PRJNA688993, SRR13354980, and SAMN17193098, respectively.
